# A Novel Protein Demonstrating Antibacterial Activity Against Multidrug-Resistant *Escherichia coli* Purified from *Bacillus velezensis* CB6

**DOI:** 10.3390/foods14071255

**Published:** 2025-04-03

**Authors:** Nan Jiang, Tajin Wang, Yue Fang, Xiaoyu Liu, Nan Dai, Hongling Ruan, Huining Dai, Lili Guan, Chengguang He, Lingcong Kong, Weixue Meng, Hongxia Ma, Haipeng Zhang

**Affiliations:** 1 College of Life Science, Jilin Agricultural University, Xincheng Street No. 2888, Changchun 130118, China; jiangnan@mails.jlau.edu.cn (N.J.); 15849982838@163.com (T.W.); 19272697663@163.com (Y.F.); 20211259@mails.jlau.edu.cn (X.L.); 20220828@mails.jlau.edu.cn (N.D.); dhn@jlau.edu.cn (H.D.); llguan@jlau.edu.cn (L.G.); hechengguang@jlau.edu.cn (C.H.); 17647055405@163.com (W.M.); 2 Changchun Shuangyang District Animal Husbandry Station, Shuangyang Street No. 586, Changchun 130600, China; 13244263160@163.com; 3 The Engineering Research Center of Bioreactor and Drug Development, Ministry of Education, Jilin Agricultural University, Xincheng Street No. 2888, Changchun 130118, China; 4 College of Veterinary Medicine, Jilin Agricultural University, Xincheng Street No. 2888, Changchun 130118, China; congwbs@126.com

**Keywords:** *Bacillus velezensis*, multidrug-resistant *Escherichia coli*, antibacterial protein, action mechanism

## Abstract

In recent years, multidrug resistance in pathogenic bacteria has become increasingly serious, causing serious harm to the livestock and poultry breeding industries and posing severe challenges to its clinical prevention and treatment; therefore, the development of new antibacterial agents is urgently needed. We previously isolated *Bacillus velezensis* CB6, which exhibits broad-spectrum antibacterial activity, from Changbaishan in China. In this study, multidrug-resistant *Escherichia coli* B2(MDR *E. coli* B2) was used as an indicator bacterium. Ammonium sulfate precipitation, dextran gel chromatography, and Diethylaminoethyl Bestarose High Performance was used to isolate antibacterial protein with strong activity against MDR *E. coli* B2. SDS–PAGE combined with liquid chromatography-mass spectrometry was used to obtain the antibacterial protein CB6-E, which has a molecular weight of 54.537 kDa. Our study found that CB6-E has a strong inhibitory effect on Gram-negative bacteria such as *Pseudomonas aeruginosa* Z1, *Salmonella* H9812, and *Shigella castellani* Z1; among them, the minimum inhibitory concentration for MDR *E. coli* B2 was 32 µg/mL. In addition, CB6-E is stable under various conditions including exposure to various temperatures, organic reagents, pH values, and proteolytic enzymes. The hemolytic activity test and cytotoxicity test also showed that CB6-E is safe. Research on antibacterial mechanisms showed that CB6-E destroys cell membranes in a dose-dependent manner and can inhibit the growth of MDR *E. coli* B2 by targeting lipopolysaccharides on the cell membrane, showing good therapeutic effects in model animals. In summary, CB6-E is a newly discovered antibacterial protein with a high therapeutic index that is safe, nontoxic, and stabile, and is expected to be an effective antibacterial agent.

## 1. Introduction

*Escherichia coli* (*E. coli*) is an opportunistic pathogen [1]. It is widely present in soil, water sources, animal feces, and the human gut, can cause various diseases such as diarrhea, urinary tract infections, life-threatening bloodstream infections, and has potential pathogenicity and strong infectivity [2]. The Centers for Disease Control and Prevention (CDC) reported a total of 131,525 cases of foodborne infections between 2009 and 2018, 49% of which were caused by pathogenic bacteria, with *E. coli* being one of the most common pathogens [3]. In recent years, the irrational use of many antibiotics has led to the emergence of multiple drug-resistant strains (MDRs) of *E. coli*, for which no specific treatments are available. At present, there is an urgent need to develop new antibiotics as alternatives to the existing ones to address the public health security caused by MDR *E. coli* infections worldwide [4]. Therefore, the search for safe and efficient new drugs against MDR *E. coli* is important.

*Bacillus*, especially *Bacillus velezensis* (*B. velezensis*), is a common type of bacteria that can produce various antibacterial substances, such as antibiotics, bacteriocins, and antimicrobial proteins, during its growth process [5,6]. Ma et al. isolated *B. velezensis* CM7-4 from seawater and showed that this bacteria produces a novel bacteriocin, PCM7-4, which exhibits broad-spectrum antibacterial activity against both Gram-positive and Gram-negative bacteria [7]. Johny et al. isolated various antimicrobial peptides from the novel marine *B. velezensis* FTL7, and its minimum inhibitory concentration (MIC) against *Listeria monocytogenes* was 2.5 µg/mL [8]. Berić et al. isolated *Bacillus licheniformis* VPS50.2 from the soil sample, and its metabolite licheniocin 50. 2 had a bactericidal effect on *Listeria monocytogenes* [9]. Currently, antibacterial substances produced by *B. velezensis* are considered effective candidate drugs for combating MDR pathogens [10].

In previous research, we isolated *B. velezensis* CB6 from the soil of the Changbaishan in China and reported that this bacterium could effectively inhibit pathogenic bacterial growth [11]. A protein with a strong inhibitory effect on methicillin-resistant *Staphylococcus aureus* (MRSA) was obtained through chromatographic column purification and mass spectrometry analysis [12]. However, recent studies have found that strain CB6 strongly inhibits Gram-negative bacteria, especially *E. coli* B2 (which is polymyxin-resistant and carbapenem-resistant) [13,14]. Therefore, in this study, *B. velezensis* CB6 was used as the background to further identify the antibacterial protein CB6-E by a protein purification method. These results indicate that the obtained antibacterial protein CB6-E has effective antibacterial activity both in vitro and in vivo as well as good stability and safety. These properties make the antimicrobial protein CB6-E a likely potential choice for antimicrobial drugs.

## 2. Materials and Methods

### 2.1. Sterile Fermentation Supernatant Preparation of Strain CB6

Compared with the previous description from our laboratory, the method for producing antibacterial active substances by the fermentation of strain CB6 was slightly improved [10]. The CB6 strain was inoculated at a concentration of 1% in 300 mL of Luria–Bertani medium (LB, Sangon Biotech Co., Ltd., Shanghai, China), the shaker temperature was 28 °C, the culture medium pH was 7.5, and the culture time was 48 h. Fermentation cultures of the CB6 strain were subsequently obtained. The cultures were centrifuged at 4 °C and 8000× *g* for 60 min, after which the supernatants were filtered through 0.22-μm membranes (Shanghai Bioleaf Biotech Co., Ltd., Shanghai, China) to obtain sterile supernatants.

### 2.2. Optimum Saturation of Ammonium Sulfate-Precipitated Crude Protein

A certain amount of ammonium sulfate was added to the sterile supernatant of the equal volume of CB6 strain, and ammonium sulfate solutions were configured with a final concentration of 50%, 60%, 70%, 80%, 90%, and 100%, respectively. At 4 °C, the mixture was stirred with a magnetic stirrer for 12 h and then allowed to rest overnight; this was then centrifuged at 8000× *g* for 60 min, then the supernatant was discarded, after which the crude protein in the precipitate was collected. The crude protein was dissolved in phosphate-buffered saline (PBS, pH 7.4), and ammonium sulfate was subsequently removed using a 500 Da dialysis bag. The crude protein mixture was filtered through 0.22-μm membranes to remove the cells. The inhibition zone of the sterile crude protein was detected via the agar diffusion method.

### 2.3. Purification of the Antibacterial Protein

The obtained fractions were purified with a dextran agarose cross-linked gel chromatographic column (the column diameter was 16 mm × 1 m and the filler was 75 PG). The fractions with different molecular weights were separated at a flow rate of 0.5 mL/min, and each single peak was collected according to the absorbance value at 280 nm using a UV detector (Cytiva, Boston, MA, USA). The activity of the fractions was verified by the agar diffusion method. In addition, the collected active fraction with antibacterial activity from the previous step was further purified using Diethylaminoethyl Bestarose High Performance (DEAE Bestarose HP, 10 mm × 300 mm) packed with a purification column (Bestchrom Biotechnology Co., Ltd., Zhengjiang, China). Briefly, the active sample was added into a DEAE Bestarose HP purification column, and the protein was eluted step-by-step using NaCl at different concentrations (0.1 M–1 M). The absorbance of every protein fraction was measured at 280 nm using a UV detector, and the activity of the protein fraction was verified via the agar diffusion method.

### 2.4. Polyacrylamide Gel Electrophoresis Analysis and Mass Spectrometry Identification

Purified antibacterial protein and electrophoresis buffer were combined and then added to the polyacrylamide gel. The voltage was adjusted to 80 V and the sample was allowed to run through the separation gel; then the voltage was adjusted to 100 V and the sample was run to the desired limit before the gel was stained using the Coomassie brilliant blue staining method. Only a single protein band was obtained, and the protein band was subsequently cut and rinsed with ddH_2_O and decolorized with decolorizing solution. Next, the strip was washed with ammonium bicarbonate and acetonitrile in turn. Finally, the glue was dehydrated until the glue turned white, and trypsin was added for enzymatic hydrolysis. After centrifugation and drying, the polypeptide samples were redissolved in Nano-LC mobile phase A (0.1% formic acid/water), bottled, and then analyzed by online liquid chromatography-tandem mass spectrometry (LC-MS/MS) (Bruker Daltonics, Bremen, Germany). Proteome Discover 2.5 software was used for processing, and a comparison analysis was made with the UniProt-*Bacillus velezensis*-uniparc_upid database.

### 2.5. MIC Determination of the Antibacterial Protein CB6-E

The MIC of the antibacterial protein CB6-E was tested according to Jia et al. [15] as previously described. Briefly, CB6-E powder was dissolved in PBS, added to the first well of a 96-well plate, and then serially diluted with a 2-fold dilution factor with PBS until the final concentration was between 0.5 and 256 µg/mL. Different indicator strains were cultivated separately in LB broth culture medium and shaken at 37 °C and 180 rpm until the OD600 reached 0.5 (Supplementary Table S1). Next, the concentration of the bacteria was adjusted to 10^5^ CFU/mL using LB medium, and an equal amount of the bacterial suspension was added to a 96-well plate containing CB6-E. After incubation at 37 °C for 16 h, the absorbance was measured at an OD value of 600 nm (Microplate reader, Tecan GENios F129004, Tecan, Salzburg, Austria). The MIC was defined as the minimum concentration at which no bacteria grew after being cultured at 37 °C for 16–20 h.

### 2.6. Stability Testing

The temperature stability of CB6-E was measured by placing an equal amount of CB6-E into multiple test tubes. Next, tubes with CB6-E were incubated in a water bath at 40 °C, 50 °C, 60 °C, 70 °C, 80 °C, 90 °C, or 100 °C for 30 min to evaluate the temperature stability of CB6-E. To test the protease sensitivity of CB6-E, CB6-E antibacterial activity was performed according to previously described methods [16]. Briefly, CB6-E was treated with an equal volume of gastric protease, catalase, trypsin, papain, or proteinase K solution (final enzyme concentration: 1 mg/mL) prepared in PBS (pH 7.0). The enzymatic reactions were incubated at 37 °C for 60 min, and then the proteolytic enzymes stability of CB6-E was evaluated. To evaluate the effect of pH on the stability of CB6-E, the same concentration of CB6-E was adjusted to pH 4–10 with hydrochloric acid or sodium hydroxide. After incubation at room temperature for 30 min, all CB6-E samples were adjusted to pH 7.0, and then the antibacterial activity was detected. To test the effects of organic reagents on the stability of CB6-E, 1% (*v*/*v*) sodium dodecyl sulfate, polyethylene terephthalate, polyoxyethylene sorbitan monopalmitate, polysorbate-80, methanol, acetone, mercaptoethanol, ethylenediaminetetraacetic acid, or isopropanol was added to the CB6-E sample, respectively, which was subsequently incubated at 37 °C for 1 h, and then the antibacterial activity was detected.

For the above experiments, untreated CB6-E was used as a control, and the antibacterial activity of the treated CB6-E samples and the control sample were measured to evaluate the factors influencing CB6-E stability.

### 2.7. Determination of Time-Kill Kinetics

*E. coli* B2 was cultivated to the logarithmic growth stage (OD_600_ = 0.5), and the bacterial concentration was adjusted to 10^5^ CFU/mL in LB medium. The bacteria were then mixed with CB6-E to prepare three CB6-E mixtures with final concentrations of 1× MIC, 2× MIC, and 4× MIC, respectively. The mixtures were subsequently shaken and cultured at 37 °C and 180 rpm for 24 h. During this period, an equal amount of liquid was taken in each group every 4 h for colony counting [17].

### 2.8. Safety Testing

To evaluate the hemolytic activity of CB6-E on sheep red blood cells, the collected sheep red blood cells were washed three times with PBS (pH 7.4) and then diluted to 1% [18]. The 1% sheep red blood cells and different concentrations of CB6-E were subsequently mixed in a tube and incubated at 37 °C for 1 h. Then, the mixtures were centrifuged at 3000× *g* for 10 min. The supernatant was subsequently transferred to a 96-well plate, and the optical density of the mixture was measured at 570 nm. To evaluate the cytotoxicity of CB6-E on RAW 264.7 and Vero cells, equal amounts of the cells were placed into 96-well plates at a density of 10^5^ cells per milliliter and incubated overnight at a concentration of 5% carbon dioxide at 37 °C. Next, 100 µL of CB6-E (2 to 1024 µg/mL) was added to 1–10 wells of the cell culture dish; 100 µL of DMEM was used as the positive control. After incubation for 16 h at 37 °C, CCK-8 solution (10%, *v*/*v*) was added into each well containing mixed cells in 96-well plates, followed by incubation at 37 °C for another 2 h. Absorbance was subsequently measured at 450 nm.

### 2.9. Evaluation of the Therapeutic Effect of CB6-E in the Galleria mellonella Model

Forty *Galleria mellonella* (*G. mellonella*) were randomly divided into five groups (n = 8 per group) and placed in disposable culture dishes containing cotton overnight at room temperature. The next day, the bacterial suspension of *E. coli* B2 after washing with sterile PBS was diluted to 1 × 10^8^ CFU/mL, 2 × 10^8^ CFU/mL, 4 × 10^8^ CFU/mL, and 1 × 10^9^ CFU/mL, respectively. Then, 10 µL of the above bacterial suspensions were injected into the left side of the abdomen of *G. mellonella* individuals from groups 1–4; 10 µL of sterile PBS was injected into the fifth group. The survival rate of *G. mellonella* was observed the next day, and the concentration of *E. coli* B2 bacterial suspension (2 × 10^8^ CFU/mL) that could cause all death of *G. mellonella* within 48 h was selected for the subsequent experiment (2 × 10^8^ CFU/mL of *E. coli* B2 bacterial suspension was the lowest dose to kill all *G. mellonella* within 48 h). After the model was established, an additional twenty-five *G. mellonella* larvae were randomly divided into five groups of five individuals that were incubated at room temperature overnight. The five groups of *G. mellonella* larvae were injected with 10 µL of *E. coli* B2 bacterial solution (2 × 10^8^ CFU/mL) at the left ventral foot. After 1 h, groups 1–3 were injected with 5 mg/kg, 10 mg/kg, or 20 mg/kg CB6-E at the right ventral foot. The fourth group of *G. mellonella* was treated with 10 mg/kg polymyxin B as a positive control group. The fifth group of *G. mellonella* was treated with 10 µL sterile PBS as a negative control group. The larvae were observed continuously for 48 h, and the survival of *G. mellonella* was recorded.

### 2.10. Evaluation of the Therapeutic Effect on Acute Peritonitis in Mice

A mouse infection model was constructed using twenty-five 4-week-old Kunming mice, half male and half female, randomly divided into five groups of five mice each and fed continuously for 7 d. After a fasting period of 12 h, 100 μL of *E. coli* B2 suspension (2 × 10^8^ CFU/mL, 4 × 10^8^ CFU/mL, 5 × 10^8^ CFU/mL, or 1 × 10^9^ CFU/mL) was injected into the right side of the abdominal cavity of mice in each group using a sterilized syringe. A fifth group was given 100 μL of sterile PBS for a 5-day survival test. *E. coli* B2 at 5 × 10^8^ CFU/mL will cause the death of all mice. Therefore, the experimental condition of the subsequent infection mouse model was 5 × 10^8^ CFU/mL of *E. coli* B2. After the model was established, fifty Kunming mice were selected and randomly divided into five experimental groups, with ten mice in each group. The mice in groups 1–5 were injected with 100 µL of the *E. coli* B2 bacterial solution (5 × 10^8^ CFU/mL) in the right side of the abdominal cavity. After 1 h, the mice in groups 1–3 were treated with 5 mg/kg, 10 mg/kg, or 20 mg/kg CB6-E After 1 h, the mice in groups 1–3 were treated with 5 mg/kg, 10 mg/kg, or 20 mg/kg CB6-E treatment. The fourth group of mice was treated with 100 µL of polymyxin B at a concentration of 0.5 mg/mL as a positive control group. The fifth group of mice was treated with 100 μL of sterile PBS as a negative control. The mice were observed continuously for 5 d, and their survival status was recorded. The animal study protocol was approved by the Laboratory Animal Ethics Committee of Jilin Agricultural University (protocol code 20230317002 and the approval date was 17 March 2023). All activities involving animal analysis, including euthanasia procedures for mice, complied with the relevant regulations and guidelines formulated by the Jilin Agricultural University Changchun Animal Care Institution.

### 2.11. Determination of Lung Tissue Lesions and Organ Bacterial Load in Mice

Fifty 4-week-old Kunming mice with a 1:1 sex ratio were randomly divided into five groups of ten mice in each group. The mice were adaptively fed for 7 d to eliminate stress. Each group of mice was given 100 µL of 5 × 10^8^ CFU/mL *E. coli* B2 bacterial suspension intraperitoneally. After infection, three groups were randomly selected and treated with 5 mg/kg, 10 mg/kg, or 20 mg/kg CB6-E. The fourth group of infected mice was treated with 100 µL of polymyxin B. The fifth group of infected mice was treated with 100 µL of sterile PBS. After 48 h, the mice were dissected under sterile conditions; the mice that had not died were euthanized, and the heart, liver, spleen, lungs, kidneys were removed, and a portion of each organ was washed, ground, and diluted with sterile physiological saline for colony counting. The remaining portion was washed with sterile physiological saline, fixed with paraformaldehyde, and sent to Sevier Biotechnology Co. Ltd. (Wuhan, China) for HE staining.

### 2.12. Effects on the Cell Membrane

The *E. coli* B2 strain was inoculated into LB medium until the OD_600_ was equal to 0.5, after which it was washed and suspended in HEPES solution containing 5 mM glucose at pH 7.4, then 10 μL of 1mM NPN solution was added to each 990 μL of bacterial suspension (10^6^ CFU/mL) and incubated at room temperature for 30 min in a dark environment. Next, 100 µL of *E. coli* B2 suspension containing NPN was added to different concentrations of CB6-E, and the outer membrane permeability of CB6-E was evaluated by detecting the fluorescence intensity of the test sample. In addition, we investigated the effect of CB6-E on the permeability of the cytoplasmic membrane of *E. coli* B2. The *E. coli* B2 strain was inoculated into LB medium and cultured until the OD_600_ reached 0.5. Next, 100 µL of *E. coli* B2 suspension was added to 10 µM propidium iodide (PI) dye, and 100 µL of CB6-E (16 µg/mL–512 µg/mL) was added to 100 µL of *E. coli* B2 suspension containing PI. A total of 100 µL of sterile PBS was added to 100 µL of *E. coli* B2 suspension containing PI as a negative control, then 100 µL of polymyxin B was added to 100 µL of *E. coli* B2 suspension containing PI as a positive control. The prepared test samples were transferred to a sterile 96-well plate. The fluorescence intensity was measured every 10 min. In addition, to investigate the effect of CB6-E on the permeability of the inner membrane of *E. coli* B2, *E. coli* B2 was added to LB medium, shaken well, and cultured in a 37 °C incubator at 180 rpm until the OD_600_ reached 0.5. Next, the bacterial suspension was centrifuged at 3000 rpm for 10 min, and the supernatant was discarded. ONPG buffer (1.5 mM) was used to suspend the bacterial cells at an OD_600_ of 0.5, then 100 µL of *E. coli* B2 suspension containing ONPG as added to 100 µL CB6-E (16 μg/mL to 512 μg/mL). The sample was transferred to a disinfected black 96-well plate, and the absorbance was measured at 420 nm at intervals of 5 min for 60 min. The detection method of biofilm formation was shown by Wang et al. [19]. Briefly, the suspension of *E. coli* B2 was cultivated to an OD_600_ of 0.5, 100 µL of the suspension was added to a 96-well dish, and the mixture was incubated at 37 °C for 24 h. 100 µL of CB6-E (16 μg/mL to 512 μg/mL) was added to each well, the mixture was incubated at 37 °C for 2 h, and then the mixture was discarded. Next, 150 µL of methanol was added, and the mixture was fixed at room temperature for 30 min. After the mixture was removed, 150 µL of crystal purple stain was added, and the mixture was incubated at room temperature for 30 min. Discard the mixture and rinse repeatedly with sterile PBS (pH = 7.4) and dry at 37 °C, then 150 uL of glacial acetic acid was added to each well where it dissolves crystal violet, after which the absorbance at 595 nm was measured.

### 2.13. ROS Release

Fluorescence staining was performed via the DCFH-DA fluorescent probe method. Briefly, the *E. coli* B2 strain was inoculated into LB medium and cultured until the OD600 reached 0.5, then DCFH-DA at a final concentration of 10 μM was added to the bacterial suspension, and the mixture was incubated at 37 °C for 30 min. A total of 100 µL of bacterial suspension containing DCFH-DA was added to 100 μL of CB6-E (16 μg/mL to 512 μg/mL). An equal volume of sterile PBS was added as the positive control, and an equal volume of hydrogen peroxide was added as the negative control.

### 2.14. Scanning Electron Microscope Observation

Scanning electron microscopy (SEM) was used to investigate the effect of CB6-E on the structure of *E. coli* B2 [20]. In brief, E. coli B2 (OD600 = 0.5) was diluted to 106 CFU/mL, added to different final concentrations of CB6-E (1 × MIC, 2 × MIC, or 4 × MIC), and cultured at 37 °C for 3 h. Untreated *E. coli* B2 was used as a control. After incubation, the bacterial suspension was removed, after which the cells were fixed overnight at 4 °C. The samples were dehydrated with an ethanol mixture, dried, and sprayed with gold prior to SEM imaging (JEOL, Hitachi, Tokyo, Japan).

### 2.15. Antibacterial Activity Assay of CB6-E and Mixtures of Different Cell Membrane Components

To detect the effects of CB6-E on the main components of the *E. coli* B2 cell membrane, 2.048 mg of purified CB6-E was weighed, mixed with 1 mL of PBS, diluted in a gradient, and added to a 96-well plate (1–10 wells). Meantime, certain concentrations of L-α-phosphatidylcholine, phosphatidylethanolamine, lipopolysaccharide, cardiolipin sodium salt, and phosphatidylglycerol were added to each experimental well and incubated together for 30 min. After *E. coli* B2 (OD_600_ = 0.5) was diluted 1000 times, an equal volume was added to the 96-well plate and incubated at 37 °C for 12 h to detect the effects of different components of the cell membrane on the antibacterial activity of CB6-E.

### 2.16. Statistical Analysis

All experiments were repeated three times. SPSS v.22.0 software was used for multivariate analysis of variance (MANOVA), followed by the Tukey test. Graph-based tests were used for data processing. The obtained data were expressed as the mean ± standard deviation. A * *p* < 0.05 or ** *p* < 0.01 was considered statistically significant, *** *p* < 0.001 or **** *p* < 0.0001 was considered high statistical significance.

## 3. Results

### 3.1. Purification of the Antibacterial Protein CB6-E

**Figure 1:**
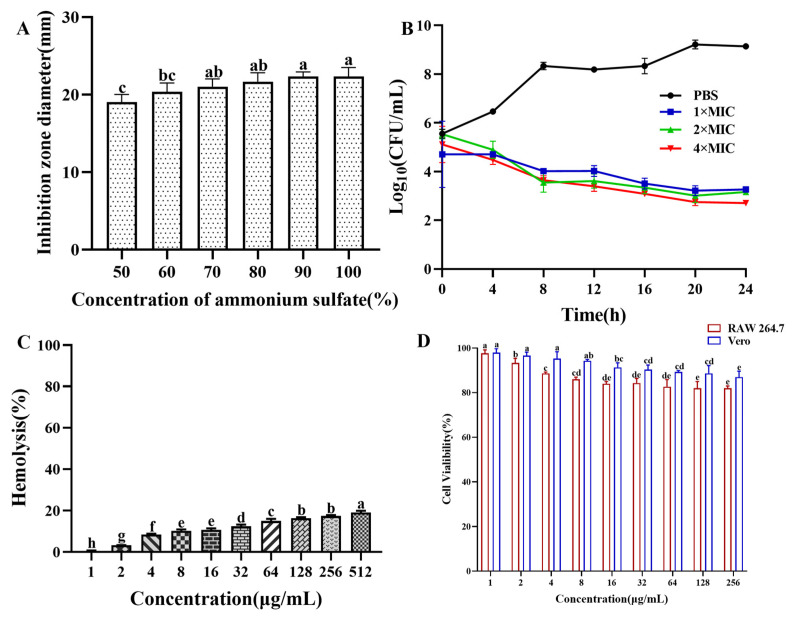
Ammonium sulfate deposition, time-kill kinetics, and safety assays of CB6-C. (**A**) Effect of ammonium sulfate with different saturations on the antibacterial activity of crude protein. (**B**) Time-kill kinetics of antibacterial protein CB6-E. (**C**) Hemolytic activity of CB6-E to the sheep red blood cells. (**D**) Cytotoxicity of CB6-E against RAW 264.7 cells and Vero cells. Different concentration groups mean a significant difference among groups (*p* < 0.05).

The saturated ammonium sulfate extraction of antibacterial crude protein showed that as the saturation of ammonium sulfate increased, the antibacterial activity of the antibacterial crude protein gradually increased. When the saturation of ammonium sulfate reached 90%, the inhibition zone was maximized. Therefore, in subsequent experiments, ammonium sulfate with a saturation of 90% was used to extract crude antibacterial protein from the fermentation supernatant of strain CB6 (Figure 1A). The antibacterial crude protein obtained was purified using a dextran agarose gel chromatography column to obtain three peaks, as shown in Supplementary Figure S1. Only the second peak showed antibacterial activity after validation via the Oxford cup drilling method. The second peak collected was concentrated using a 3 kDa ultrafiltration tube and purified using a DEAE Bestarose HP exchange column to obtain five peaks. The results indicated that peak 4 had a good antibacterial effect in a 0.2 M NaCl solution. The diameter of the inhibition ring of *E. coli* B2 was 22 mm. Molecular weight determination was performed via SDS–PAGE. As shown in Supplementary Figure S2, only a protein band with a molecular weight of approximately 54 kDa was obtained, whose molecular size was preliminarily identified as the target band. Analysis of the antibacterial protein via LC–MS/MS revealed a molecular size of 54.537 kDa, which was a similar molecular size to the protein obtained by polyacrylamide gel electrophoresis (Supplementary Figures S2–S4). The protein sequence similarity analysis (UniProt database, https://www.uniprot.org) showed that the antibacterial protein CB6-E shared a 94.8% sequence identity with *Bacillus subtilis* 168 vegetative catalase (KatA; accession number: P26901; molecular weight: 54.791 kDa) and was designated as the antibacterial protein CB6-E.

### 3.2. Antibacterial Activity of CB6-E by MIC Assay

The MICs of CB6-E against various pathogens were determined using the broth microdilution method. As shown in Supplementary Table S1, the MICs of CB6-E against *Acinetobacter baumannii* C1, *Klebsiella pneumoniae* T1, *Enterococcus faecalis* T1, and *Staphylococcus aureus* ATCC25923 were greater than or equal to 256 µg/mL. The MIC for *Shigellagastellani* Z1 was 64 µg/mL, and the MICs for *E. coli* B2, *Pseudomonas aeruginosa* Z1, and *Salmonella* H9812 were 32 µg/mL.

### 3.3. Stability Assay

The analysis of temperature, pH, enzyme, and chemical reagent stabilities revealed that as the temperature gradually increased, the antibacterial activity of CB6-E gradually decreased. After heating at 90 °C for 30 min, it retained 62% of the activity compared with the untreated group. After 30 min of treatment at 100 °C, 40% antibacterial activity was still detected. In addition, the antibacterial activity of CB6-E was measured in different pH environments. As the pH gradually increased, the antibacterial activity gradually increased. When the pH was 7, the maximum activity of CB6-E was 100%. However, as the pH approached alkalinity, the antibacterial activity decreased again. The antibacterial activity of CB6-E after hydrolysis by different proteolytic enzymes was measured, and after 60 min of peroxidase, trypsin, pepsin, and papain hydrolysis, CB6-E still retained 73% activity. After 30 min of chemical reagent treatment, the antibacterial activity of CB6-E remained at 65% (Supplementary Table S2).

### 3.4. Determination of Time-Kill Kinetics

As shown in Figure 1B, the number of *E. coli* B2 bacteria decreased significantly at different concentrations of CB6-E (1 × MIC, 2 × MIC, or 4 × MIC) over 24 h but were not completely eliminated. These findings indicate that CB6-E can significantly reduce the survival rate of *E. coli* B2 but cannot completely kill *E. coli* B2.

### 3.5. Safety Assay

The hemolytic effect of CB6-E on sheep red blood cells is shown in Figure 1C. As the content of CB6-E increased, the hemolysis rate slightly increased. At 16 µg/mL, the hemolysis rate of sheep red blood cells was approximately 10%. The results of the cell safety experiments showed that high concentrations of CB6-E had no significant inhibitory effect on mouse RAW 264.7 or Vero cells. At a concentration of 256 µg/mL, the viabilities of mouse RAW 264.7 and Vero cells were over 85% and 90%, respectively (Figure 1D). These findings indicate that CB6-E has good biocompatibility at appropriate doses.

### 3.6. Evaluation of the Therapeutic Effect on G. mellonella

**Figure 2:**
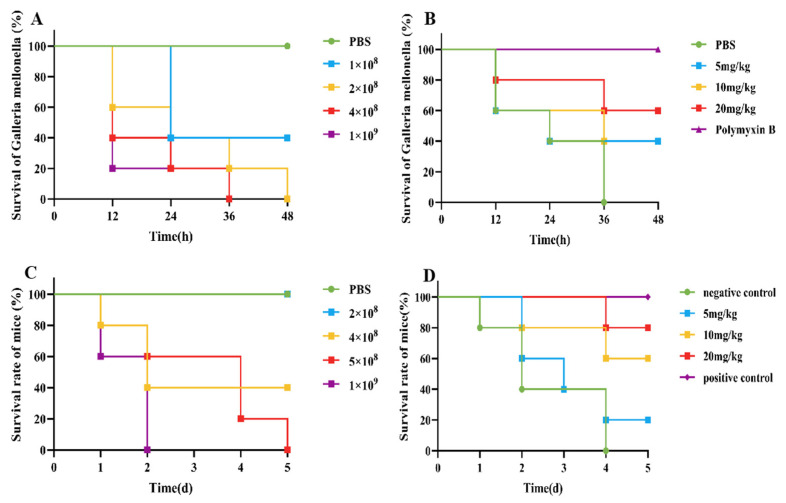
CB6-E was efficient in preventing infections of *E. coli* B2. (**A**) Survival rates of the *G. mellonella* infected by *E. coli* B2 (n = 8 per group). (**B**) Survival rate of *G. mellonella* infected with *E. coli* B2 after treatment with CB6-E. (**C**) Survival rates of the mice infected by *E. coli* B2 (n = 5 per group). (**D**) Survival rate of mice infected with *E. coli* B2 after treatment with CB6-E.

An infection model for *G. mellonella* was established, as shown in Figure 2A. Compared with those in the control group, when 10 µL of 2 × 10^8^ CFU/mL *E. coli* B2 bacterial suspension was given intraperitoneally, *G. mellonella* were all died in 48 h. Therefore, 10 µL of 2 × 10^8^ CFU/mL *E. coli* B2 was selected for the infection of *G. mellonella*. After an infection model of *G. mellonella* was established, the survival rate of *G. mellonella* within 48 h was 40% after treatment with 5 mg/kg and 10 mg/kg CB6-E, whereas the survival rate of *G. mellonella* within 48 h was 60% after treatment with 20 mg/kg CB6-E (Figure 2B).

### 3.7. Evaluation of the Therapeutic Effect on Mice

**Figure 3:**
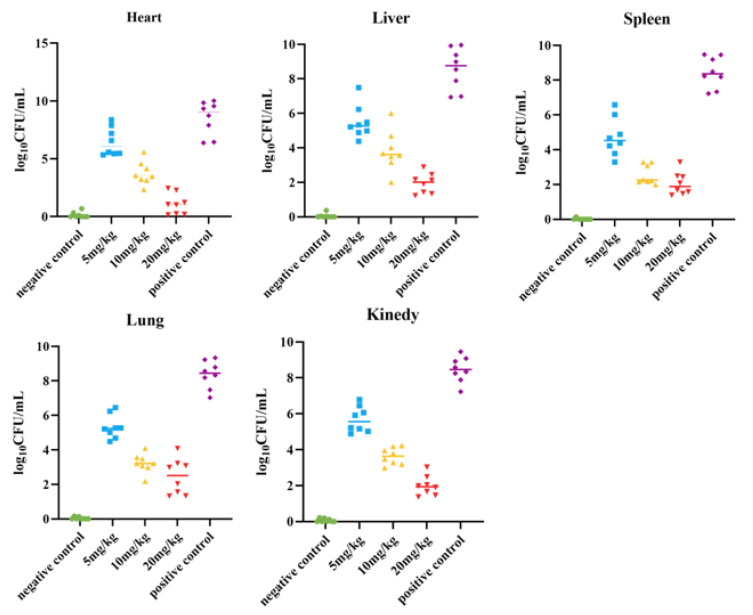
Determination results of bacterial load in the organs (heart, liver, spleen, lung, kidney) of mice.

**Figure 4:**
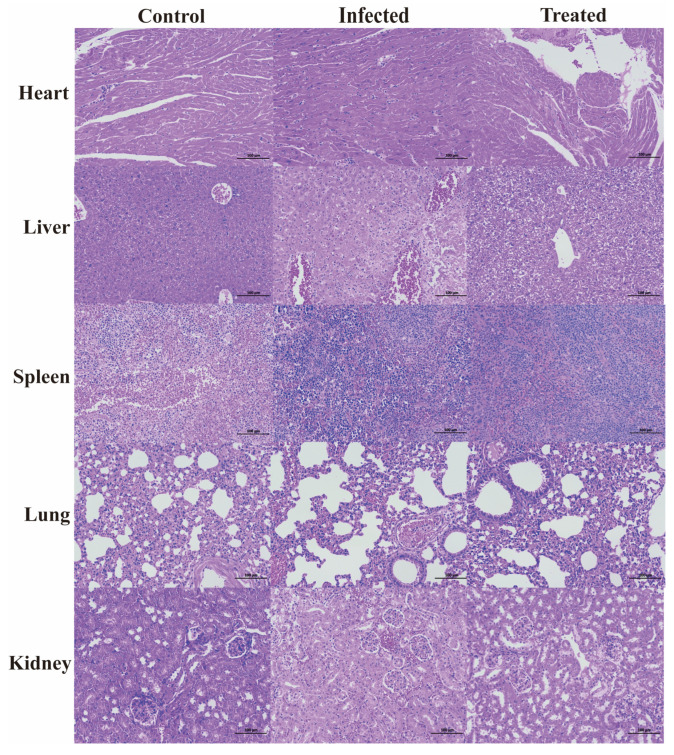
Histologic analysis of the tissues in mice using hematoxylin–eosin staining (×100).

The survival rate of the mice was tested, and a mouse peritonitis infection model was established. When 100 µL of 5 × 10^8^ CFU/mL *E. coli* B2 bacterial suspension was given intraperitoneally, the survival rate of the mice was 60% within 2 d, 20% within 4 d, and all mice died within 5 d (Figure 2C). Therefore, 100 µL of 5 × 10^8^ CFU/mL *E. coli* B2 was selected to infect the mice. Figure 2D shows the therapeutic effect on mice treated with the antimicrobial protein CB6-E for 5 d. After treatment, the survival rate of the experimental group significantly improved, and the survival rate of the 20 mg/kg antimicrobial protein CB6-E group was the highest. In addition, compared with that of the negative control mice, mice treated with the antimicrobial protein CB6-E presented a significant reduction in the bacterial organ burden in Figure 3. The results of the HE staining of mouse organs are shown in Figure 4. Mice infected with *E. coli* B2 exhibited severe hepatic vacuolization and significant inflammatory infiltration. The heart, spleen, lungs, and kidneys showed hemorrhagic spots, punctate necrosis, dark red cytoplasm, interstitial bleeding, and granular chromatin changes. After treatment with CB6-E, the pathological changes in various tissues and organs of infected mice were alleviated. These results indicate that CB6-E has a good therapeutic effect on organ damage in mice.

### 3.8. Determination of Cell Membrane Permeability

**Figure 5:**
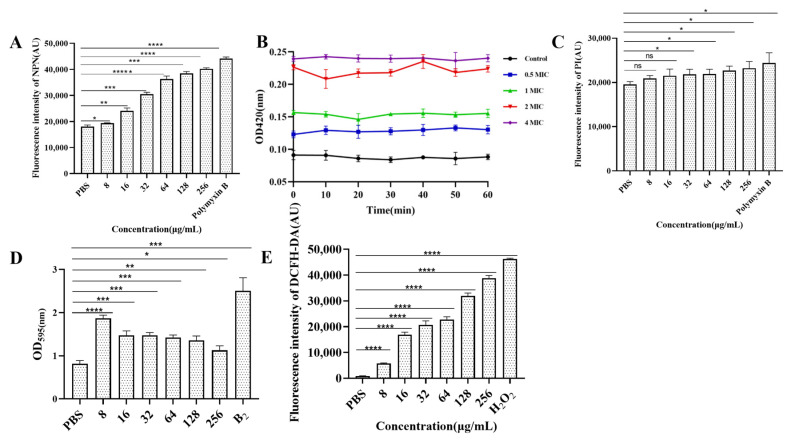
Effect of antibacterial protein CB6-E on the cell membrane of *E.coli* B2. (**A**) The effect of the outer membrane permeability. (**B**) The effect of the inner membrane permeability. (**C**) The effect of the cytoplasmic membrane. (**D**) The results of biofilm formation in *E.coli* B2 after treatment with antibacterial protein CB6-E. (**E**) The results of ROS in *E.coli* B2 cells after treatment with antibacterial protein CB6-E. Statistical significance is indicated as follows: * *p* < 0.05; ** *p* <0.01; *** *p* < 0.001; **** *p* < 0.0001, ***** *p* < 0.00001.

The results of outer membrane permeability are shown in Figure 5A. Compared with that of PBS, the outer membrane permeability of *E. coli* B2 increased with increasing CB6-E content. The results of inner membrane permeability are shown in Figure 5B. A 2 × MIC of CB6-E significantly increased the permeability of the *E. coli* B2 membrane. By detecting changes in fluorescence intensity, the cytoplasmic membrane permeability can be detected, as shown in Figure 5C. Compared with that of the PBS control group, CB6-E did not significantly change the cytoplasmic membrane permeability of *E. coli* B2. In addition, the results concerning the role of CB6-E in *E. coli* B2 biofilms are shown in Figure 5D. CB6-E significantly reduced the ability of *E. coli* B2 to create bacterial biofilms.

### 3.9. ROS Measurement

The ROS measurements are shown in Figure 5E. CB6-E significantly increased the accumulation of intracellular ROS in *E. coli* B2, and as the amount of CB6-E increased, the intracellular ROS content also increased. This result indicates that the dose of CB6-E is positively correlated with the intracellular ROS content in *E. coli* B2.

### 3.10. Scanning Electron Microscope Observations

**Figure 6:**
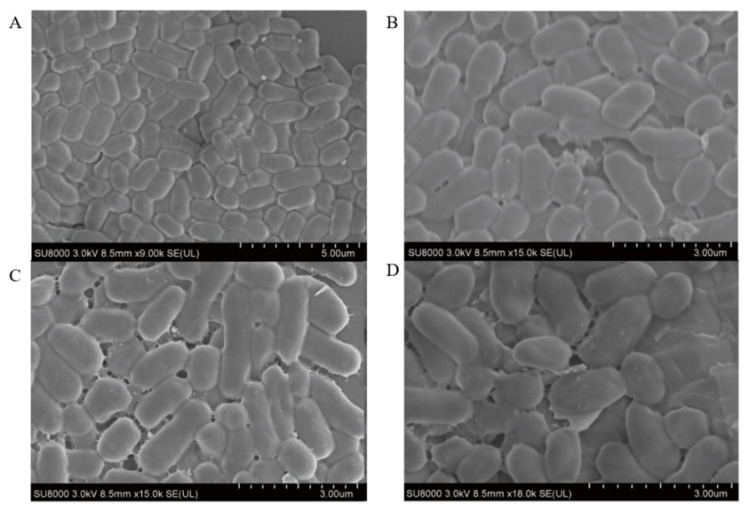
Scanning electron microscopy images of *E. coli* B2 cells treated with antibacterial protein CB6-E. (**A**) Untreated control *E. coli* B2 cells. (**B**) *E. coli* B2 cells treated with CB6-E (1 × MIC). (**C**) *E. coli* B2 cells treated with CB6-E (2 × MIC). (**D**) *E. coli* B2 cells treated with CB6-E (4 × MIC).

The morphological changes in *E. coli* B2 treated with CB6-E were observed using SEM. The results are shown in Figure 6. After treatment with CB6-E at 4 × MIC, the degree of adhesion to the surface of *E. coli* B2 increased, and the cells were greatly damaged. The surface of *E. coli* B2 contracted irregularly, with many cracks. In contrast, the *E. coli* B2 group without CB6-E treatment showed an integral cell structure, and the bacterial surface was smooth and full. The experimental results demonstrate that CB6-E can damage and lyse *E. coli* B2 cells.

### 3.11. Evaluation Antibacterial Activity of CB6-E and Mixtures of Different Cell Membrane Components

As shown in Supplementary Table S3, adding different concentrations of phosphatidylglycerol and cardiolipin esters to bacteria did not significantly affect the MIC of *E. coli* B2. After the addition of phosphatidylethanolamine and L-α-phosphatidylcholine, the MIC of CB6-E against *E. coli* increased from 32 to 64 μg/mL. After the addition of lipopolysaccharide, the MIC of CB6-E against *E. coli* B2 increased from 32 μg/mL to 128 μg/mL. The results show that *E. coli* B2 could target lipopolysaccharides on the cell membrane of CB6-E.

## 4. Discussion

In recent years, with the long-term and improper use of antibiotics, pathogenic bacteria have become increasingly resistant to antibiotics, leading to the emergence of Gram-negative bacteria such as MDR *E. coli.* In health care institutions, the spread of MDR *E. coli* can pose a serious threat to patients, especially those with weakened immune systems [21]. *B. velezensis* is a ubiquitous spore-forming bacterium belonging to the genus *Bacillus*. It is widely distributed in natural environments such as soil, dust, and water sources and can also be found in food [22]. Due to the high tolerance of *B. velezensis* to the environment, the antibacterial substances produced by *B. velezensis* usually have good stability [23]. This enables the antibacterial substances produced by *B. velezensis* to maintain their activity under different environmental conditions, making them more valuable for research. This study found that *B. velezensis* CB6 has good antibacterial activity against MDR *E. coli* B2 under certain fermentation conditions. CB6-E was purified using ammonium sulfate precipitation, a dextran agarose cross-linked chromatography column, and a DEAE Bestarose HP exchange column. A single target band was obtained by polyacrylamide gel electrophoresis, and the molecular weight of CB6-E was 54.537 kDa based on LC–MS/MS. A UniProt database search revealed that the antibacterial protein had a 94.8% sequence identity similar to the vegetative catalase (KatA) protein of *Bacillus subtilis* 168 (accession number: P26901). Previous research has shown that the KatA protein induces abnormal hyphal elongation and conidial swelling and rupture in fungi, significantly inhibiting anthracnose development [24]. However, there have been no reports on its ability to inhibit bacterial growth. Consequently, this study is the first to analyze the antibacterial mechanism of CB6-E against MDR *E. coli* B2.

Currently, an important factor limiting the clinical use of antibacterial proteins is their stability. We examined the effects of temperature, pH, proteolytic enzymes, and chemical reagents on the stability of CB6-E. Our results showed that CB6-E exhibited the best stability at 37 °C, which is consistent with previous research findings [10]. In addition, after incubation at 90 °C for 60 min, the antibacterial activity of CB6-E remained at 62% (Supplementary Table S2), indicating that CB6-E has excellent heat resistance, which facilitates its use in storage and transportation and improves its prospects for applications in other fields [25]. In addition, CB6-E has strong pH tolerance, with an optimal pH of 7. It maintains 65% antibacterial activity in strongly acidic environments (pH 4.0) and 65% antibacterial activity in strongly alkaline environments (pH 10). This suggests that CB6-E has broad pH stability, making it a candidate for antibacterial drugs. CB6-E also has excellent enzymatic stability, although it is highly sensitive to protease K. In addition, organic solvents such as methanol and isopropanol do not affect the antibacterial activity of CB6-E. In summary, the antimicrobial protein CB6-E exhibits a variety of stable characteristics such as high-temperature resistance, acid and alkali resistance, enzyme resistance, and organic reagent resistance. These properties make it a potential candidate for an antimicrobial agent.

On this basis, time-kill kinetics were used to investigate the effects of the CB6-E content and duration of action on the proliferation rate of *E. coli* B2. This study found that when CB6-E was used at 1 × MIC, 2 × MIC, or 4 × MIC, it significantly inhibited the growth of *E. coli* B2 but could not completely eliminate it (Figure 1B). As a new type of antibacterial drug, the hemolytic ability and cytotoxic effect of CB6-E are important indicators for evaluating its safety [26]. Therefore, the hemolytic activity and cytotoxicity of CB6-E were tested. At all detected concentrations, CB6-E showed low hemolytic activity and cytotoxicity, suggesting that at a given dose, the antimicrobial protein CB6-E is safe and nontoxic to animals (Figure 1C,D). To test the in vivo bactericidal effect of CB6-E on *E. coli* B2, infection models were established in *G. mellonella* and mice. After treatment with different concentrations of CB6-E, the survival rate of infected *G. mellonella* increased (Figure 2B). After a mouse model of acute peritonitis was established, the bacterial loads in the tissues and organs were decreased and the symptoms of acute peritonitis infection were alleviated after treatment with different concentrations of CB6-E (Figure 3 and Figure 4). These results showed that CB6-E had a good therapeutic effect on acute peritonitis in mice.

Previous studies have shown that ROS in the body are crucial for sterilization [27]. Our preliminary research revealed that CB6-E can increase the intracellular ROS levels in *E. coli* B2, thereby exacerbating cell death, but its molecular mechanism is still unclear. The results of the cell membrane permeability assay showed that CB6-E significantly affects the permeability of the inner and outer membranes of *E. coli* B2 cells. Moreover, SEM analysis revealed that after treatment with CB6-E, the bacterial cells adhered, wrinkled, and sustained damage. We speculate that this phenomenon may be related to the action of CB6-E on bacterial cell membranes. Gram-negative bacteria have a very rich cell membrane content, including phosphatidylcholine, phosphatidylethanolamine, lipopolysaccharide, phosphatidylglycerol and cardiolipin, which plays a crucial role in maintaining the stability of cell membranes [28,29,30]. Experiments with antibacterial activity of CB6-E and mixtures of different cell membrane components revealed that CB6-E mainly acts through lipopolysaccharides to influence *E. coli* B2 proliferation. Taken together, these findings suggest that CB6-E can enter the cell mainly by destroying lipopolysaccharide on the cell membrane, causing intracellular ROS accumulation and subsequently inhibiting the growth of *E. coli* B2.

## 5. Conclusions

In summary, through a series of protein separation techniques, we isolated a novel antimicrobial protein, CB6-E, with strong antibacterial activity against *E. coli* B2 and an MIC of 32 µg/mL. Biological activity tests were conducted on CB6-E, and the results showed that it is stable and safe. CB6-E targets lipopolysaccharides on the membrane surface of *E. coli* B2, increasing the intracellular ROS accumulation and inducing cell death. These findings suggest that CB6-E has the potential to become an effective antimicrobial agent.

## Data Availability

The original contributions presented in the study are included in the article/Supplementary Material, further inquiries can be directed to the corresponding authors.

## Supplementary Materials

The following supporting information can be downloaded at: https://www.mdpi.com/article/10.3390/foods14071255/s1, Figure S1: Elution profile of antibacterial protein CB6-E; Figure S2: SDS-PAGE of antibacterial protein CB6-E; Figure S3: Secondary spectrums of peptide segments produced from CB6-E; Figure S4: Amino acid sequence of CB6-E; Table S1: The MIC of antibacterial protein CB6-E against pathogenic bacteria; Table S2: Stability of CB6-E after treating with temperature, pH, proteolytic enzymes, and organic reagent; Table S3: Effects of additional different cell membrane components on the anti- *E. coli* B2 activity of CB6-E.
