# Supplementary material for: A Novel Protein Demonstrating Antibacterial Activity Against Multidrug-Resistant Escherichia coli Purified from Bacillus velezensis CB6
Source: Foods. 2025 Apr 3;14(7):1255. doi: 10.3390/foods14071255 (PMC11988598; doi:10.3390/foods14071255)
Supplement: Supplementary file 1 [file foods-14-01255-s001.zip › foods-3504528-supplementary.pdf]

Article

A novel protein demonstrating antibacterial activity against multidrug-resistant *Escherichia coli* purified from *Bacillus velezensis* CB6

Nan jiang [1], Tajin Wang [1], Yue Fang [1], Xiaoyu Liu [1], Nan Dai [1], Hongling Ruan [2], Huining Dai[1, 3], Lili Guan [1, 3], Chengguang He[1, 3], Lingcong Kong [4], Weixue Meng [1], Hongxia Ma[1, 3, 4*], Haipeng Zhang[1, 3*]

Supplementary Figure

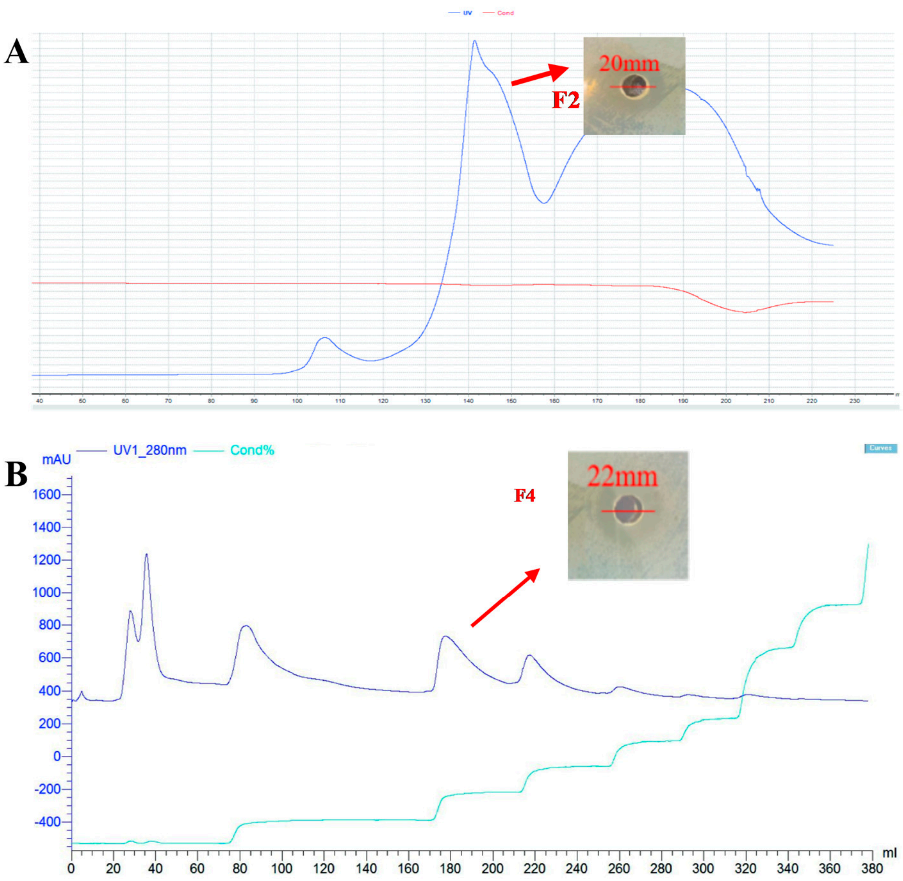

Supplementary Figure S1. Elution profile of antibacterial protein CB6-E. (A) Dextran agarose cross-linked gel chromatographic column elution profile of antibacterial protein CB6-E. (B) DEAE Bestarose HP elution profile of antibacterial protein CB6-E.

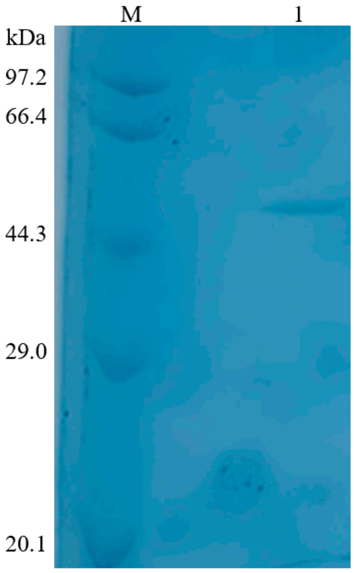

Supplementary Figure S2. SDS-PAGE of antibacterial protein CB6-E. Lane M: High molecular marker, lane 1：Stained of the gel showing purified antibacterial protein CB6-E.

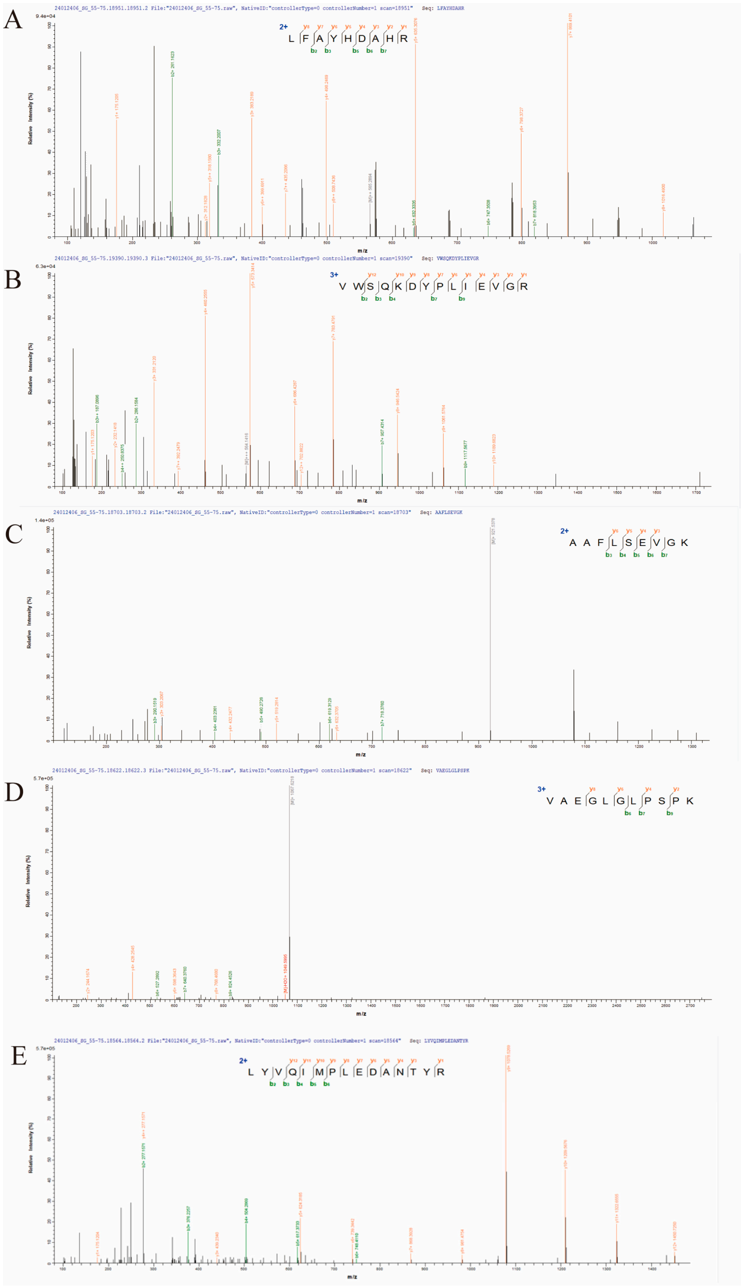

Supplementary Figure S3. Secondary spectrums of peptide segments produced from CB6-E.

1-50	:	MSSNKLTTSWGAPVGDNQNSMTAGDRGPALIQDVHLLEKLAHFNRERVPE
51-100	:	RVVHAKGAGAHGYFEVTNDVTKYTKAAFLSEVGKRTPLFIRFSTVAGELG
101-150	:	SSDTVRDPRGFAVKFYTEEGNYDIVGNNTPVFFIRDAIKFPDFIHTQKRD
151-200	:	PRTHLKNPTAVWDFWSLSPESLHQVTILMSDRGIPATLRHMHGFGSHTFK
201-250	:	WTNDKGEGVWIKYHFKTEQGVKNLDVNTAAKIAGENPDYHTEDLFNAIEN
251-300	:	GDFPAWKLYVQIMPLEDANTYRFDPFDVTKVWSQKDYPLIEVGRMVLNRN
301-350	:	PENYFAEVEQATFSPGTLVPGVDVSPDKMLQGRLFAYHDAHRYRVGANHQ
351-400	:	ALPINRSRNEVKNYQRDGQMRFDDNGGRSVYYEPNSFGGPKESPEDKQAA
401-450	:	YPVSGFADSVSYNHHDHYTQAGDLYRLMSEEERARLVANIVSAMKPVEKE
451-481	:	EIKLRQIGHFYKADPEYGRRVAEGLGLPSPK

Supplementary Figure S4. Amino acid sequence of CB6-E

Supplementary Table

Supplementary Table S1. The MIC of antibacterial protein CB6-E against pathogenic bacteria

Gram reaction and strains	Source/Reference	Broth medium	MIC (μg/mL)
**Gra-negative bacteria**			
Escherichia coli B2	stored in our laboratory	LB	32
Escherichia coli K88	stored in our laboratory	LB	32
Pseudomonas aeruginosa Z1	stored in our laboratory	LB	32
Salmonella H9812	stored in our laboratory	LB	32
Shigella castellani Z1	stored in our laboratory	LB	64
Acinetobacter baumannii C1	stored in our laboratory	LB	>256
Klebsiella pneumoniae T1	stored in our laboratory	LB	>256
**Gra-positive bacteria**			
Staphylococcus aureus	ATCC25923	LB	256
Methicillin-resistant Staphylococcus aureus (MRSA)	stored in our laboratory	LB	256
Enterococcus faecalis T1	stored in our laboratory	LB	>256

Supplementary Table S2. Stability of CB6-E after treating with temperature, pH, proteolytic enzymes, and organic reagent

Temperature	Time (min)	Residual activity (%)
Control	30	100 ± 0.030
40°C	30	100 ± 0.042
50°C	30	95 ± 0.023
60°C	30	89 ± 0.020
70°C	30	74 ± 0.031
80°C	30	71 ± 0.034
90°C	30	62 ± 0.028
100°C	30	40 ± 0.040
**pH**	**Time (min)**	**Residual activity (%)**
Control	30	100 ± 0.020
4	30	65 ± 0.044
5	30	70 ± 0.035
6	30	96 ± 0.033
7	30	100 ± 0.020
8	30	95 ± 0.023

9	30	80 ± 0.026
10	30	65 ± 0.028
**Proteolytic enzymes**	**Time (min)**	**Residual activity (%)**
Control	60	100 ± 0.022
Peroxidase	60	82 ± 0.015
Pepsin	60	73 ± 0.024
Trypsin	60	80 ± 0.035
Papain	60	75 ± 0.027
Proteinase K	60	48 ±0.023
**Chemical Reagents**	**Time (min)**	**Residual activity (%)**
Control	30	100 ± 0.030
Sodium Dodecyl Sulfonate	30	70 ± 0.031
Polyethylene terephthalate	30	68 ± 0.024
Polyoxyethylene sorbitan Monopalmitate	30	65 ± 0.029
Polysorbate 80	30	70 ± 0.030
Methanol	30	96 ± 0.021
Acetone	30	100 ± 0.026
2-Mercaptoethanol	30	95 ± 0.027
Ethylenediamine tetraacetic Acid	30	80 ± 0.034
Isopropanol	30	65 ± 0.038

Supplementary Table S3. Effects of additional different cell membrane components on the anti- *E. coli* B2 activity of CB6-E

Antimicrobial proteins	MIC(μg/mL)				
	L-αPhosphatidylcholine	Phosphatidyl-ethanolamine	Lipopoly-saccharide	Phosphatidylglycerol	Cardiolipin
	64	64	128	32	32
